# Integration of a constraint-based metabolic model of *Brassica napus* developing seeds with ^13^C-metabolic flux analysis

**DOI:** 10.3389/fpls.2014.00724

**Published:** 2014-12-19

**Authors:** Jordan O. Hay, Hai Shi, Nicolas Heinzel, Inga Hebbelmann, Hardy Rolletschek, Jorg Schwender

**Affiliations:** ^1^Biological, Environment and Climate Sciences Department, Brookhaven National LaboratoryUpton, NY, USA; ^2^Department of Molecular Genetics, Leibniz-Institut für Pflanzengenetik und KulturpflanzenforschungGatersleben, Germany

**Keywords:** loopless flux balance analysis, ^13^C-metabolic flux analysis, central metabolism, carbon partitioning, constraint-based reconstruction and analysis

## Abstract

The use of large-scale or genome-scale metabolic reconstructions for modeling and simulation of plant metabolism and integration of those models with large-scale omics and experimental flux data is becoming increasingly important in plant metabolic research. Here we report an updated version of bna572, a bottom-up reconstruction of oilseed rape (*Brassica napus* L.; Brassicaceae) developing seeds with emphasis on representation of biomass-component biosynthesis. New features include additional seed-relevant pathways for isoprenoid, sterol, phenylpropanoid, flavonoid, and choline biosynthesis. Being now based on standardized data formats and procedures for model reconstruction, bna572+ is available as a COBRA-compliant Systems Biology Markup Language (SBML) model and conforms to the Minimum Information Requested in the Annotation of Biochemical Models (MIRIAM) standards for annotation of external data resources. Bna572+ contains 966 genes, 671 reactions, and 666 metabolites distributed among 11 subcellular compartments. It is referenced to the *Arabidopsis thaliana* genome, with gene-protein-reaction (GPR) associations resolving subcellular localization. Detailed mass and charge balancing and confidence scoring were applied to all reactions. Using *B. napus* seed specific transcriptome data, expression was verified for 78% of bna572+ genes and 97% of reactions. Alongside bna572+ we also present a revised carbon centric model for ^13^C-Metabolic Flux Analysis (^13^C-MFA) with all its reactions being referenced to bna572+ based on linear projections. By integration of flux ratio constraints obtained from ^13^C-MFA and by elimination of infinite flux bounds around thermodynamically infeasible loops based on COBRA loopless methods, we demonstrate improvements in predictive power of Flux Variability Analysis (FVA). Using this combined approach we characterize the difference in metabolic flux of developing seeds of two *B. napus* genotypes contrasting in starch and oil content.

## Introduction

Large-scale metabolic network reconstruction, modeling, and simulation is becoming increasingly important in plant systems biology and metabolic engineering (Schwender, 2011; Sweetlove and Ratcliffe, 2011; Collakova et al., 2012; Seaver et al., 2012; De Oliveira Dal'molin and Nielsen, 2013; Sweetlove et al., 2014). Metabolic networks for constraints-based metabolic modeling have been reconstructed for plants including *Arabidopsis* (Poolman et al., 2009; Williams et al., 2010; Dal'molin et al., 2011; Mintz-Oron et al., 2012; Cheung et al., 2013; Arnold and Nikoloski, 2014), barley (Grafahrend-Belau et al., 2009, 2013; Rolletschek et al., 2011), *Chlamydomonas* (Boyle and Morgan, 2009; Manichaikul et al., 2009; Chang et al., 2011; Dal'molin et al., 2011; Kliphuis et al., 2012), corn (Saha et al., 2011), oilseed rape (Schwender, 2008; Hay and Schwender, 2011a,b; Pilalis et al., 2011; Schwender and Hay, 2012; Borisjuk et al., 2013), rice (Lakshmanan et al., 2013; Poolman et al., 2013), C4 plants (De Oliveira Dal'molin et al., 2010b), and CAM plants (Cheung et al., 2014). Draft models have been generated for seven additional plants with sequenced genomes (Seaver et al., 2014). The methods framework being generally used has been summarized as COnstraint-Based Reconstruction and Analysis (COBRA) (Thiele and Palsson, 2010). In constraint-based (CB) analysis, networks are simulated using mathematical constraints related to mass conservation (reaction stoichiometry), thermodynamics (reaction directionality) and various physiological/experimental data.

A metabolic reconstruction of an organism can be understood as a highly structured representation of biochemical, genomic, and physiological information which allows derivation of a mathematical model useful for constraint-based analysis (Thiele and Palsson, 2010). Standard operating procedures that describe the reconstruction process in detail and standards for quality control (e.g., confidence scores to quantify the level of biological evidence for reactions) and model testing have been developed (Thiele and Palsson, 2010). Together with model features like mass and charge balanced reactions and definition of gene-protein-reaction (GPR) associations a high-quality standard in model reconstruction is defined.

A continuously growing extensive suite of COBRA methods have gained rapid acceptance, providing diverse tools for model refinement, modeling of genetic perturbations, or characterization of the solution space of large-scale models (Lewis et al., 2012). Since CB modeling often results in a solution space with multiple possible flux distributions, methods to narrow down the flux solution space are in demand (Reed, 2012). For example, experimentally determined metabolic flux ratios can be imposed as additional constraints for flux balance analysis methods (Choi et al., 2007; Mcanulty et al., 2012). Imposition of loop-law constraints (Schellenberger et al., 2011a; Noor et al., 2012) shrinks the solution space by removing solutions that contain thermodynamically infeasible loops.

In recent years different methods have been developed aiming at using gene expression data to derive tissue specific models from generic organism specific networks, or to improve the predictive power of FBA (Blazier and Papin, 2012; Lewis et al., 2012; Machado and Herrgard, 2014). Chang et al. (2011) validated the reaction network in a metabolic reconstruction of *Chlamydomonas* based on transcritomic data. Mintz-Oron et al. (2012) used proteomic data to generate tissue specific models from their *Arabidopsis thaliana* metabolic reconstruction. In order to explore to what extent changes in flux in central metabolism can be related to changes in gene expression, a number of studies integrated differential gene expression data with metabolic flux predictions (Akesson et al., 2004; Daran-Lapujade et al., 2004; Bordel et al., 2010).

In this paper we report the revision of a CB model of seed development in the oilseed crop *Brassica napus*, bna572 (Hay and Schwender, 2011b), hereafter referred to as bna572+. We integrate bna572+ with transcriptome data to improve confidence in the model's ability to represent a developing seed. Carrying on with a biomass-guided, bottom-up approach to reconstruction, bna572+ has additional metabolic subsystems and seed-relevant biomass components. Bna572+ features standardized annotations of external data resources (Le Novere et al., 2005; Juty et al., 2012), quality assurance and control (Thiele and Palsson, 2010), COBRA toolbox compatibility (Schellenberger et al., 2011b; Ebrahim et al., 2013), mass and charge balanced reactions as well as GPR associations that account for subcellular compartmentalization. Furthermore, we compare two *B. napus* genotypes differing in seed oil accumulation based on ^13^C-Metabolic Flux Analysis (^13^C-MFA) of cultured developing embryos and derive two genotype specific CB-models. Using ^13^C-MFA derived flux constraints and the COBRA loopless algorithm (Schellenberger et al., 2011a), the solution space could be substantially reduced.

## Materials and methods

### *In-vitro* embryo culture and biomass composition measurements

Plants of oilseed rape accessions 3170 and 3231 (both maintained by the IPK Genebank) were grown in 30 cm plastic pots in a general purpose soil mix (Pro Mix BX, Premier Horticulture Inc., Quakertown, PA) under constant conditions (15°C nights, 20°C days; 16 h day, 400 μE m^−2^ s^−1^). Developing embryos were dissected aseptically about 20 days after flowering and grown in a liquid medium at 20°C under continuous light (50 μmol m^−2^ s^−1^). Cultures were kept in tissue culture flasks with vented seal cap (CytoOne T75 #CC7682-4875, USAScientific, Ocala, FL, USA), containing 13 ml of liquid medium and four embryos per flask. The liquid growth medium contained 20% (w/v) polyethylene glycol 4000, sucrose (80 mM), glucose (40 mM), Gln (35 mM), and Ala (10 mM). In labeling experiments, sucrose and glucose were partly substituted with ^13^C-labeled analogs so that [1-^13^C]- and [U-^13^C_6_]- mono and disaccharide hexose moieties are overall at 8.125 and 10 mol% of total hexose moieties, respectively (sucrose, 59 mM; [1-^13^C_fructosyl_]-sucrose, 6.5 mM; [1-^13^C_glucosyl_]-sucrose, 6.5 mM; [U-^13^C_12_]-sucrose, 8 mM; glucose, 32.75 mM; [1-^13^C]-glucose, 3.25 mM; [U-^13^C_6_]-glucose, 4 mM). ^13^C-labeled substrates were purchased from Omicron Biochemicals (http://www.omicronbio.com/). Inorganic nutrients were as described in Schwender and Ohlrogge (2002) with KNO_3_ and NH_4_NO_3_ being omitted. pH was adjusted to 5.8 using KOH. Growth medium was sterilized using 0.22-μm sterile vacuum filter units (Stericup; Millipore). After 10 days of culture, embryos were harvested, rapidly rinsed with NaCl solution (0.33 M), and after determination of fresh weight embryos were frozen in liquid nitrogen and stored at −80°C.

The biomass composition of embryo material was determined after organic solvent extraction and liquid:liquid fractionation into a chloroform soluble (lipid), methanol/water soluble (polar), and insoluble cell polymer fraction as reported earlier (Lonien and Schwender, 2009). The resulting biomass composition data and biomass derived fluxes used for ^13^C-MFA are listed in Table S2A.

In addition, extraction and analysis of soluble metabolites were done according to previously described protocols (Rolletschek et al., 2011). In brief, frozen embryos were ground to a powder with steel beads and extracted with precooled (−20°C) methanol/chloroform/water. Acephate was added during extraction as an internal standard. After centrifugation the supernatant was filtered with centrifugal filters and distributed in three aliquots.

To determine metabolite concentrations, we applied hydrophilic interaction chromatography (HILIC) using the instrument Ultimate 3000 (Dionex, Idstein, Germany) coupled to triple quadrupole mass spectrometer (MS; ABSciex, Darmstadt, Germany). The amino propyl column (Luna NH2, 250 × 2 mm, particle size 5 μm, Phenomenex, Torrance, CA) was used with varying eluent gradients, adjusted to either negative or positive scan mode of the MS. Some metabolites were detected via ion chromatography (ICS-3000-System, Dionex, Idstein, Germany) coupled to the same MS in negative ion mode. All chromatographic conditions, MS settings and ion traces used for multiple reaction monitoring in the various detection modes were exactly as listed in Rolletschek et al. (2011). Compound identities were verified by mass and retention time match to authenticated standards (Sigma, Germany). External calibration was applied using authenticated standards to determine metabolite concentrations (nmol/g dry weight of tissue).

Among the 57 analyzed free metabolites quantified by LC/MS, sucrose, Gln, malate and citrate are the most abundant ones, accounting for 80% (w/dw) of the free metabolites. In order to account for fluxes into these four major free metabolites, the weight of the methanol/water soluble fraction was divided among those metabolites.

### ^13^C-metabolic flux analysis

For both genotypes ^13^C-labeling signatures were measured by mass spectrometric analysis as outlined before (Lonien and Schwender, 2009). Altogether fractional ^13^C-enrichment was determined by GC/MS in 18 biomass derived metabolites (33 molecular fragments, 163 peaks). Particular amino acids (derived from hydrolysis of cell pellet fraction), glycerol and fatty acids (derived from the lipid fraction), and glucose (derived from starch in cell pellet fraction) were analyzed (Table S2F).

Flux analysis was performed using the 13CFLUX2 computational toolbox (Weitzel et al., 2013) using the Brookhaven Linux Cluster (Scientific Linux 4). The network of central metabolism is defined by 25 free exchange fluxes, 13 free net fluxes as well as 21 biomass effluxes (Tables S2B–E) which are derived from biomass composition of the different genotypes (Table S2A). In addition, proxy reactions (Lonien and Schwender, 2009; Williams et al., 2010) were used to allow the model to adjust for the effect of isotopic disequilibrium in labeled polymers caused by preexisting unlabeled biomass. This adds one additional free net flux (Table S2E, net equalities section in model file). In order to find the global optimum 500 simulations were run with random start values for the free fluxes. The theoretical χ^2^-value was calculated to test the goodness of fit. Based on the general model information, the number of degrees of freedom is 91 (163 modeled MS signals + 21 biomass flux measurements; less 33 molecular fragments, 35 free net fluxes and 25 free exchange fluxes). With a 90% confidence level, χ^2^-value should be less than 108.65 (χ^2^_*90%,91*_ = 108.65) to pass the statistical test. From the 500 random start optimizations, 28 and 43 solutions had a sum of squared residuals smaller than the χ^2^-critical value for genotype 3231 and 3170, respectively. For each genotype, the final flux estimates were derived by averaging the 10 best fit flux sets. Then statistical uncertainty in the best fit flux values (Table S2F) was determined with a Monte Carlo approach, by 100 times random re-sampling of the mass spectrometric data and flux measurements (Gaussian distribution; 13CFlux2 function “perturb”). Each time a best fit was selected from 20 random start optimizations.

The resulting flux distributions for genotypes 3231 and 3170 were used to constrain the flux space in the large scale stoichiometric metabolic model (bna572+) by transferring 9 out of 13 possible degrees of freedom. In the following we shortly summarize the necessary considerations about the definition of flux states, precision of flux estimates and the treatment of possible incompatibilities between the models based on network stoichiometries. Table S2B and C define mappings between reactions of both models, which we used to transfer flux values. A flux solution in the ^13^C-model is defined by a total of 35 free net fluxes (Table S2H). Twenty one of these don't need to be transferred since they are biomass dependent fluxes, which are derived independently in bna572+ based on the same biomass composition data (see below under paragraph 2.7). One free net flux (“vAla_bm”) in the ^13^C-model, used for adjustment to isotopic disequilibrium by preexisting unlabeled biomass, is excluded as well since it is not relevant for bna572+. This leaves 13 free net fluxes that define a flux state transferrable to bna572+. However, the flux solutions obtained in ^13^C-MFA have limited accuracy and precision, dependent on network structure, substrate labeling, measurement configuration and measurement errors. We therefore inspected the covariance matrix of the free net flux data generated by Monte Carlo statistics. This indicated that most of the statistical uncertainty is given by vGPT, vPPT, and vG6PDHp. We therefore excluded these three weakly determined free fluxes, leaving 10 free fluxes to be transferred to bna572+ (vuptAla, vupt0, vuptGln, vRub_p, vPEPC_c, vPyr_cp, vICDH, vPyr_cm, vME_c, vME_p). Two of them (vMEc, vMEp) were relatively close to zero and were therefore defined as knock-out constraints in bna572+ (**Table 2**).

Finally, we recognized that transfer of flux estimates from the ^13^C-model to bna572+ can cause unbalanced flux states in bna572+, in particular if absolute flux values are to be transferred. For example, in the ^13^C-model the uptakes of Ala and Gln are not derived based on tracing nitrogen, but based on unlabeled amino acid substrates isotopically diluting intermediates derived from ^13^C-labeled sugar substrates. While nitrogen is not balanced in the ^13^C-model, it is comprehensively balanced in bna572+. By transfer of Ala and Gln uptake rates from the ^13^C-model even very small numerical deviations from the nitrogen balance would render the bna572+ stochiometry non-solvable. As shown by this example, transfer of absolute net fluxes was avoided by formulation of flux states in the ^13^C-model by using flux ratios (**Table 2**; Table S2H).

### Bna572+: metabolic network reconstruction revision and availability

For metabolic network reconstruction of bna572+, we developed an Excel-based metabolic knowledge database. The core of the reconstruction is available in Excel format (Tables S1A–C), along with associated worksheets (Table S1D–F), COBRA-compatible Systems Biology Markup Language (SBML) (File S1), a demonstration of how the core Excel content is translated into SBML using LibSBML (Bornstein et al., 2008) and Python (File S2), and a Matlab COBRA test script (File S3).

### Update of biochemical reaction equations

Biochemical reactions and metabolites data are in Table S1B,C. Notation for subcellular compartmentalization is defined as “no compartment” (“u”), “cell wall apoplast” (“a”), “cytosol” (“c”), “mitochondrial intermembrane space” (“i”), “plastid thylakoid lumen” (“l”), “mitochondrial matrix” (“m”), “inner mitochondrial membrane” (“n”), “plastidic stroma” (“p”), “plastid thylakoid membrane” (“t”), “peroxisome” (“x”), and “external environment” (“e”). Charge balancing was carried out according to compartment pH and the chemicalize.org database. The cytosol was assumed to be at pH 7.5 and plastid stroma and mitochondrial matrix compartments more alkaline (pH 8.0 and 7.8, respectively). Reaction and metabolite data was written to SBML using a Python script (File S2).

### Revision of gene-protein-reaction associations

Gene-Protein-Reaction associations (GPRs) are detailed in Table S1A. Bna572 GPRs (Hay and Schwender, 2011b) were revised to account for subcellular compartmentalization. To do this, we used the SUBA3 database (Tanz et al., 2013). Reconstruction of multiprotein complexes is based on Aracyc (Mueller et al., 2003) and additional literature (Table S1F).

In Table S1A a numerical association scheme is used to relate genes to reactions. Each association is encoded by up to three numbers separated by periods (e.g., 1.1.1). The first number specifies the reaction, the second a complex, and the third a subunit. Genes with the same numerical association code are related to each other by “or” Boolean logic. If genes associate with the same complex (i.e., second number is the same), but different subunits (i.e., third number different), then an “and” association is made. The numerical associations were translated into COBRA-compatible GPR strings and written to SBML using a Python script (File S2).

To compare *Arabidopsis* genes encoding for central metabolism enzymes to *B. napus* orthologs, we obtained predicted protein sequences of the diploid progenitor species of *B. napus*, *Brassica rapa*, and *Brassica oleracea* from genomic databases (http://www.ocri-genomics.org/bolbase/, Liu et al., 2014; ftp://ftp.jgi-psf.org/pub/compgen/phytozome/v9.0/Brapa/assembly/, Wang et al., 2011). Altogether, 86777 predicted protein sequences from the *B. rapa* and *B. oleracea* genomes were combined into a BLAST searchable database.

### Confidence scoring and integration of gene expression data

Confidence scores were used to justify the inclusion of reactions in the reconstruction. The scoring system (Table S1E) is based on literature, external databases, and published *B. napus* omic*s* data sets referenced to *Arabidopsis* gene identifiers. The default score for a reaction was zero. A score of “1” was used if the reaction is included for modeling and simulation purposes only. “2” was given at the level of physiological evidence, for example, a spontaneous non-enzymatic reaction and for *B. napus* chloroplast proteome (Demartini et al., 2011). “3” was used for biochemical evidence in Brassicaceae species other than *B. napus* using BRENDA (Schomburg et al., 2013), or for developing *B. napus* embryo transcriptome (Troncoso-Ponce et al., 2011) and *B. napus* embryo plastid proteome data (Demartini et al., 2011). “4” was the highest score awarded, and used for biochemical evidence (e.g., enzyme activity assayed) in *B. napus* according to the literature or BRENDA.

GPRs were evaluated using the gene-reaction association rules in the COBRA model structure (field name “rules”) and a true/false presence/absence call for gene expressed based on expression profiling in developing *B. napus* embryos (Demartini et al., 2011; Troncoso-Ponce et al., 2011).

### Genotype-specific constraints-based metabolic modeling

Metabolic models of *B. napus* genotypes 3170 and 3231 were generated from the Systems Biology Markup Language (SBML) encoding of bna572+ (version: bna20140218T082916.xml; File S1). The COBRA toolbox 2.0.5 (Schellenberger et al., 2011b) for MATLAB (version R2013a, http://www.mathworks.com), libSBML (version libSBML-5.8.0-libxml2-x86) was used for metabolic modeling. We include a test script (File S3) for simulation in MATLAB using COBRA. The reader is referred to a protocol (Hay and Schwender, 2014) for more details on using the model with COBRA.

Flux ratios were constrained according to flux estimates from the ^13^C-MFA models. Flux ratios defined in the ^13^C-MFA model are formulated using bna572+ flux names given by reaction projections from the ^13^C-MFA model to bna572+ (Table S2C). The flux ratio equations were added as rows to the stoichiometric matrix (S) and right-hand side (b), as has been carried out in other studies (Hay and Schwender, 2011a,b; Mcanulty et al., 2012). To accomplish this, we modified the COBRA function addRatioReaction (Thiele and Palsson, 2010) to handle complex flux summations (see function addFluxRatioReaction in File S3).

The biomass equations are expressed in terms of millimole demands for the synthesis of one gram of biomass. They were written according to the biomass composition determined for each genotype. RNA (rna[u]) and DNA (dna[u]) contents (0.1% w/w each), nucleic acid composition, and amino acid composition were modeled after bna572 (Hay and Schwender, 2011b). New compounds found in bna572+ include alpha-tocopherol (atoco[p]; isoprenoid biosynthesis), beta-sitosterol (ss[c]; sterol biosynthesis), sinapine (sincho[c]; phenylpropanoid and choline biosynthesis), and two kaempferol triglucosides (ktg[c] and sinktg[c]; flavonoid biosynthesis). The abundances of these minor components in seed biomass were estimated from the literature. Phenylpropanoids/flavonoids were accounted as small fractions of the insoluble cell wall fraction: ktg[c] 0.01% (w/w), sinktg[c] 0.04% (w/w) and sincho[c] 0.62% (w/w) (Fang et al., 2012). Small amounts of alpha-tocopherol and beta-sitosterol were attributed to the lipid fraction: atoco[p] 0.01% (w/w oil) (Goffman and Mollers, 2000); ss[c] 0.1% (w/w oil) (Gunstone, 2001). Note that the genotype-specific biomass equations (Table S1Q) are not found in the SBML, but instead are implemented in the COBRA MATLAB script (File S3).

### Flux variability analysis

The COBRA toolbox 2.0.5 (Schellenberger et al., 2011b) and TOMLAB CPLEX solver package (version 12.3, http://tomopt.com) were used for metabolic simulation. Flux variability analysis (FVA), a two level optimization, was used to quantify minimum and maximum values for all reactions. Minimization of the molar sum of all substrate uptakes and light flux has been introduced before as the primary linear objective applicable to various simulated substrate combinations (Hay and Schwender, 2011b). This objective was developed as a rationalization of photoheterotrophic metabolism of cultured embryos growing with multiple possible organic substrates (Hay and Schwender, 2011a,b). In short, our objective function is to be applied in the primary optimization and in effect determines total carbon uptakes and net CO_2_ release in agreement with an experimentally given global carbon balance. In this study the carbon balance is given with the ^13^C-MFA data (total carbon substrate uptakes relative to CO_2_ efflux, ratio 7, **Table 2**). In secondary optimizations solutions that gave 100% of the optimal value were obtained. The COBRA setting for tolerance of optimal value accuracy (the COBRA variable CBT_LP_PARAMS.objTol) was applied globally at 10^−10^ μmol/h. FVA was implemented by setting the “allowLoops” option of the “fluxVariability” function to “false.” To improve the performance of loopless FVA, it was performed only on loop reactions. Loopless FVA sometimes took longer than usual (usually no more than one loop reaction per FVA), and the solver logged the message “Consider using CPLEX node files to reduce memory usage.” Minimum and maximum fluxes returned by COBRA's loopless FVA algorithm had to be adjusted because “fluxVariability” underestimates them by a factor of 1000 for some unknown reason (see e.g., line 0218 of the source code). For some reason the maximum flux of reactions #552–553 for flux ratio-constrained 3170 was sensitive to CBT_LP_PARAMS.objTol; for these optimizations the tolerance was increased to 10^−9^ μmol/h.

The variability type (Hay and Schwender, 2011b) was used to qualitatively characterize the flux intervals predicted with FVA. For classification of reactions according to variability types, absolute flux values ≤ 10^−7^ μmol/h and those > 100 μmol/h were judged zero and unbounded/infinite (loop reaction), respectively.

Projected flux variability analysis was implemented in COBRA according to previously reported methods using toolboxes for metabolic modeling (Hay and Schwender, 2014). The only difference is that “allowLoops” was set to “false” when using “optimizeCbModel.” This method was also used as an independent approach to verify the output of loopless FVA using “fluxVariability.”

By comparing flux variability analyses between genotypes, reactions were classified according to responsiveness type. Responsiveness type characterizes the qualitative difference in a flux interval at high oil relative to low oil (Schwender and Hay, 2012). Each symbolic responsiveness type is four-partite (composed of the low-oil genotype variability type, high-oil genotype variability type, minimum flux response, and maximum flux response). A flux is termed oil responsive if its magnitude increases with oil content. If a flux magnitude decreases with oil content it is termed starch responsive. In the responsiveness type, an “r” was used to denote flux directionality reversal. For classification of reactions according to responsiveness types, absolute flux differences > 10^−7^ μmol/h were judged significant.

Using GPR associations of bna572+ as a scaffold, FVA was integrated with transcriptome data as detailed in the companion paper (Schwender et al., 2014).

### Topological analysis, mapping and random sampling of metabolic network

Correlated reaction subsets were determined by using “sampleCbModel” and “identifyCorrelSets” in the COBRA toolbox. Blocked reactions were tested for using min/max optimization of each flux in the unconstrained network. For random sampling of the genotype-specific models, the COBRA function “sampleCbModel” was used with default settings. Sampling was performed after constraining lower and upper flux bounds of all reactions in the model according to the optimal flux space defined by loopless FVA. For each sampled vector the projections of bna572+ reactions onto the ^13^C-MFA network (Table S2C) were used to derive a sampled set of ^13^C-MFA fluxes. The Euclidian distance between the ^13^C-MFA results and the projected sampled flux set was determined in each case.

For integration of FVA data with a reaction map we used COBRA “readCbMap” and “drawFluxVariability.” The map file is provided as a text file and svg (Files S4, S5). Loops were identified by computing type III extreme pathways using the Expa toolbox (Bell and Palsson, 2005). Input and output files for Expa are provided (File S6) as well as the python/COBRApy script used to generate them (File S7). A loop reaction was deemed reversible if it could carry loop flux in both directions.

## Results

### History of Bna572

**Figure 1:**
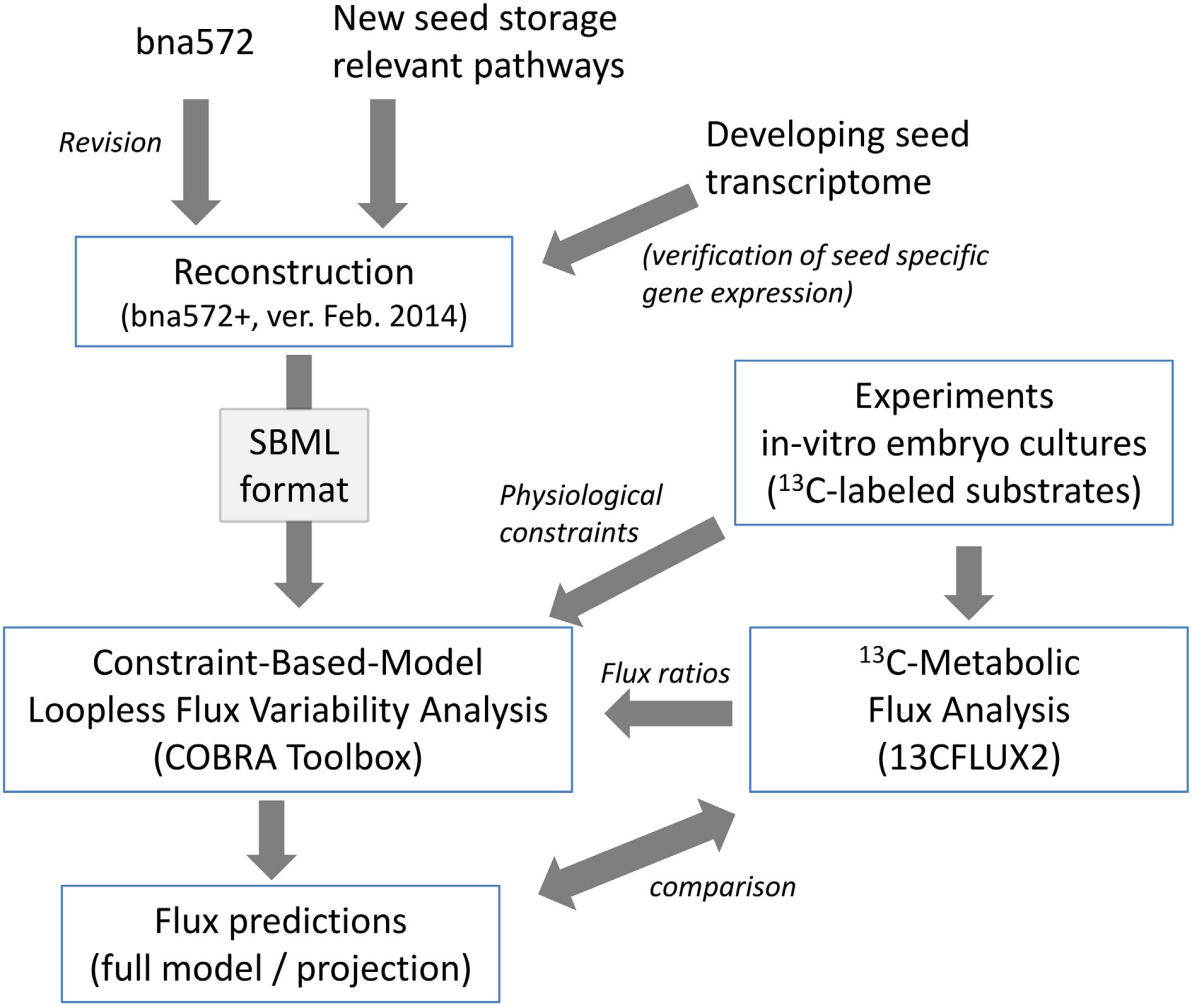
**Illustration of procedures for reconstruction of bna572+ and integration of ^13^C-MFA data**.

Bna572, a large-scale stoichiometric model with 572 reactions, has been developed to model storage compound synthesis in developing seeds of *B. napus*, using linear optimization and integrating physiological inputs generated with *in-vitro* cultivation of developing embryos (Hay and Schwender, 2011a,b). Using bna572 we modeled seed composition by *in silico* exploration of a tradeoff between two biomass components (Schwender and Hay, 2012), and explored the tissue specific metabolic heterogeneity that can be observed in the embryo while developing in planta (Borisjuk et al., 2013). Bna572 works with commonly used toolboxes for metabolic modeling and simulation (Hay and Schwender, 2014). Here we report revision and extension of bna572 (Hay and Schwender, 2011b) according to current standards for genome-scale metabolic reconstructions (bna572+). Using two genotype accessions of *B. napus* that differ in seed composition (Table 1), we demonstrate integration of flux information obtained from ^13^C-Metabolic Flux Analysis (Table 2) with loopless FVA. Our workflow is delineated as a flowchart shown in Figure 1.

**Table 1:** **Biomass composition of *B. napus* genotypes, measured for *in-vitro* cultured embryos of the two accessions 3170 and 3231**.

	**3170 [%w/dw]**	**3231 [%w/dw]**
**EMBRYO DRY WEIGHT COMPOSITION**		
Lipid	40.26±1.87	30.36±2.61
Protein	14.14±3.36	14.11±3.81
Starch	17.26±1.99	23.24±1.04
Cell wall	12.29±3.52	10.30±3.33
Gln	00.69±0.49	01.12±0.46
Malate	01.17±0.20	02.24±0.35
Citrate	04.18±0.55	05.41±1.94
Sucrose	10.01±3.99	13.21±5.91
**FATTY ACID COMPOSITION IN LIPID FRACTION (% OF TOTAL**		
**WEIGHT)**		
C16 (C_16_H_31_O_2_)	05.15±0.29	05.22±0.26
C18:0 (C_18_H_35_O_2_)	01.45±0.01	01.42±0.14
C18:1 (C_18_H_33_O_2_)	24.59±2.33	28.83±1.22
C18:2 (C_18_H_31_O_2_)	17.36±0.46	14.43±0.88
C18:3 (C_18_H_29_O_2_)	12.34±0.57	10.79±0.99
C20:0(C_20_H_39_O_2_)	00.76±0.09	00.86±0.07
C20:1(C_20_H_37_O_2_)	13.14±0.59	12.05±0.14
C22:0(C_22_H_43_O_2_)	00.72±0.09	00.51±0.05
C22:1(C_22_H_41_O_2_)	24.47±1.93	25.89±2.61

*More details on monomer composition of protein, starch and cell wall fractions and computation of biomass fluxes see Table S2A*.

**Table 2:** **Flux ratio- and flux constraints used to define constraint-based models for genotypes 3170 and 3231**.

**Flux ratio or flux equation (^13^C-MFA)**	**Values derived from ^13^C-MFA**		**Flux ratio or flux equation (bna572+)**	**Comment**
	**3170**	**3231**		
r1 = 2 vRub_p/(2 vRub_p + vGAPDH_c + vGAPDH_p)	0.45±0.08	0.38±0.08	r1 = (2 RuBisC_p + RuBisO_p)/(2 RuBisC_p + RuBisO_p + PGK_p + PGK_c + ALDH_c)	Flux through RubisCO shunt relative to glycolysis^a^
r2 = vuptAla/vuptGln	1.14±0.41	1.24±0.33	r2 = Ex_ala_a/Ex_gln_a	Uptake of amino acids
r3 = vPyr_cp/vPK_p	0.15±0.08	0.21±0.07	r3 = H_PYR_sym_c_p / PK_p	Plastidic pyruvate
r4 = vME_m/vPyr_cm	0.21±1.30	0.12±2.2	r4 = ME_m/PTP_c_m	Mitochondrial pyruvate
r5 = vPK_c/vPEPC_c	3.02±1.22	2.92±0.94	r5 = (PK_c - PPDK_c)/(PEPC_c - PPCK_c)	Cytosolic PEP
r6 = vICDH/vPyr_cm	−0.14±0.45	−0.18±0.41	r6 = (IDH_c + IDH_p + IDH_m)/PTP_c_m	Citrate cycle flux relative to pyruvate flux into mitochondria
r7 = (3 vuptAla + 5 vuptGln + 6 vupt0 + 6 vuptU)/vCO2_out	5.30±0.37	6.06±0.50	r7 = (5 Glu_H_sym_a_c + 3 Ala_H_sym_a_c + 4 Asn_H_sym_a_c + 5 Gln_H_sym_a_c + 6 Fru_H_sym_a_c + 6 Glc_H_sym_a_c + 12 SUT_a_c)/Ex_co2_a	Total substrate uptakes (carbon flux) relative to CO_2_ efflux
vME_c	0.28±0.07	0.27±0.08	ME_c = 0	Low values in the ^13^C -domain are constrained to zero
vME_p	0.24±0.23	0.16±0.05	ME_m = 0	

a *(Hay and Schwender, 2011a)*.

### Bna572+ database and revision of Bna572

Following standards from current protocols for generating Biochemically, Genetically, and Genomically (BiGG) structured knowledgebases (Schellenberger et al., 2010; Thiele and Palsson, 2010), we use an Excel workbook with three core worksheets containing information about genes (Table S1A), reactions (Table S1B), and metabolites (Table S1C). Each section contains external data resource annotations. Since a fully annotated genome of *B. napus* is not available, *Arabidopsis* is used as the reference genome. The genes worksheet (Table S1A) lists 962 *Arabidopsis* genes grouped based on associations with reactions. Definition of sub-cellular compartmentation of enzymes and reactions is documented as a decision process based on literature and mining the SUBA3 database (Tanz et al., 2013). The reactions worksheet includes notes on the heuristic decision rules (Hay and Schwender, 2011b) used to define reaction directionality.

Table 3 summarizes updates that have been made by comparing bna572 to bna572+, the version we report here. To distinguish between reconstruction improvements and expansion, the Table also includes information for just the first 572 reactions of the revision (bna572 revised). Improvements include new literature annotations, revised gene identifiers and EC numbers (Tables S1G,H), and revised/expanded subsystem assignments. Following MIRIAM guidelines for external resource annotation (Le Novere et al., 2005), we now exclusively use Pubmed identifiers, Digital Object Identifier, or International Standard Book Number (ISBN) for literature references. Metabolites have KEGG compound ID annotations and identifiers including CHEBI, Pubchem, Inchi, and Smiles. Metabolites have charges, and charged and neutral formulae are distinguished. To this end, we now organize the metabolite worksheet of the database (Table S1C) by compartment-specific metabolite pools. Using the COBRA toolbox, we evaluated reactions for mass and charge balancing. Because of the lack of careful stoichiometric balancing of water and protons, bna572 has 130 mass imbalanced reactions. The revised bna572 has only 8 mass imbalances, which remain for photons and storage compounds for which limited data are available.

**Table 3:** **Content statistics for the revision and expansion of bna572**.

	**bna572**	**bna572_revised**	**bna572+**
Genes	558	854	962^a^
Genes removed	n/a	37	37
Compartment-specific metabolites^b^	579	579	666
Metabolites (metabolite species)	376	376	457
Compartment-specific metabolites	579	579	666
Unique KEGG metabolite IDs	227	295	371
Unique Chebi metabolite IDs	0	270	333
Unique Pubchem metabolite IDs	0	296	372
Unique Inchi metabolite annotations	0	252	319
Compartments	11	11	11
Biomass components	8	8	15
Reactions	572	572	669^a^
Reactions with associated genes	367	463	543
Reactions with “AND” associated genes	0	66	66
Unique EC numbers	216	217	279
Unique references	76	91	98
Unique subsystems	35	84	93
Mass imbalanced reactions	130	8	9
Charge imbalanced reactions	n/a	2	3
Reactions with a confidence score	0	572	669
Reactions with changed stoichiometric constraints	n/a	6	6
Reactions with changed thermodynamic constraints	n/a	0	0
Blocked reactions^c^	0	0	0

a *Does not include 4 genes and 2 reactions added to genotype specific models (see **Table 4**)*.

b *Compartmentalized metabolite pools*.

c *When there is an exchange reaction for myricetin[c]*.

#### Reference genome

The use of *Arabidopsis* as a reference genome for gene annotation is based on the high similarity in protein sequences between *Arabidopsis* and *Brassica* (Cheng et al., 2012). To further substantiate this assumption we performed an exploration of orthologous relationships. We aligned all bna572+ associated predicted protein sequences against all nuclear encoded predicted protein sequences for *B. rapa* (Liu et al., 2014) and *B. oleracea* (Wang et al., 2011), the two genetic progenitor species of *B. napus* (Protein BLAST 2.2.27+, *e*-value cut-off of 10^−50^; Altschul et al., 1997). Among the blast hits were 4102 unique *Brassica* protein sequences, which were in turn aligned against the complete *A. thaliana* proteome (Protein BLAST 2.2.27+), resulting in 762 bidirectional best hits. Ninety five percentage of these alignments had >80% identity in amino acid residues. Using TargetP (version 1.1), localization prediction was identical between tentative orthologs in 88% of all cases and for about 5% the difference in prediction was between mitochondrial and plastidic localization. We conclude that proteins encoding for central metabolism are highly conserved between *B. napus* and *A. thaliana* not only on a protein sequence level but also with regards to subcellular localization.

Revision of gene associations resulted in 100 of the original reactions in bna572 receiving gene associations, while for four reactions existing gene associations were removed based on compartment specific information. An example of revisions made is the compartmentation of the Pentose Phosphate Pathway (PPP). Bna572 features a complete cytosolic PPP (reactions 512–514; 548–551; Table S1B) in parallel to the plastidic one. Yet, it has been reported that in the *A. thaliana* genome only plastid isoforms of transketolase and transaldolase are known (Eicks et al., 2002). So far no cytosolic isoforms have been found in *A. thaliana* and recently transketolase (AT2G45290, AT3G60750) has been localized to the chloroplast based on *in vivo* protein tagging (YFP) (Rocha et al., 2014). In our revision the reactions TKT1_c, TKT2_c, and TALDO_c have no genes associated. Reviewing gene associations of other relevant reactions suggests that in the cytosol ribulose 5-phosphate, produced by oxidative decarboxylation of glucose 6-phosphate, can be converted to xylulose 5-phosphate (ribulose-phosphate 3-epimerase, RPE_c) and then transported into the plastid by the xylulose 5-phosphate translocator (Pi_p_X5P_c_anti) where it can be further metabolized by the plastidic PPP (Table S1B). The lack of cytosolic transketolase and transaldolase isoforms seems to be confirmed in the genomes of *B. oleracea* and *Brassica rapa*. We found six complete sequences highly similar to the *Arabidopsis* transketolase sequences (Bol030169, Bol037547, Bol045625, Bra004898, Bra007555, Bra014473). All of them include conserved transit peptide sequences that are predicted to localize to the chloroplast (TargetP).

#### New pathways

Taking a pathway-by-pathway approach to the reconstruction process, the model was expanded to 669 reactions, 458 metabolites (672 compartmented metabolite pools), and 962 genes (Table 3). Bna572+ has five new *Brassicaceae*-relevant components in the biomass and nine new subsystems, including isoprenoid (methylerythritol phosphate and mevalonate pathways), alpha tocopherol, sterol, phenylpropanoid, flavonoid, and choline biosynthesis. The new subsystems are presented as a COBRA reaction map (File S5).

To assess whether the revision could impact any of our formerly reported simulations, stoichiometry and reaction directionality of bna572 were compared against bna572+. Disregarding the balances of waters and protons (except for proton motive force) and the new biomass equations, the first 572 reactions of bna572+ are identical to bna572. In other words, no changes were made to the stoichiometric and thermodynamic directionality constraints of the first 572 reactions of bna572+.

During expansion of the model, a flavonoid biosynthesis subnetwork was added including the biosynthesis of 3′5′ hydroxylated flavones (myricetin, dihydromyricetin, pentahydroxyflavanone) by reactions 635, 637, 638, 639, 641 (Table S1B). Literature indicates that *Arabidopsis* and *Brassica* do not synthesize 3′5′-hydroxylated anthocyanins (Sheahan et al., 1998; Falginella et al., 2010; Fang et al., 2012), and genes encoding for flavanoid 3′,5′-hydroxylase (EC 1.14.13.88) appear to be absent from the *Arabidopsis* genome (Falginella et al., 2010) and from *B. rapa* (Guo et al., 2014). Note that therefore the synthesis of 3′5′ hydroxylated flavones was inactivated in the genotype specific model simulations (Table 4), which is a modification to be implemented after import of the SBML model file into COBRA. Likewise, reactions reported to exist and to be critical for seed development, but not yet included in the SBML, are added directly to the COBRA model in the script. These are cytosolic NADP-dependent malic enzyme (Wheeler et al., 2005) and plastidic NAD-malate dehydrogenase (Scheibe, 2004), which has recently been found to be essential for embryo viability in *Arabidopsis* during seed development (Beeler et al., 2014; Selinski et al., 2014). Both of these reactions are expressed in *B. napus* developing seeds according to transcriptome data (Troncoso-Ponce et al., 2011).

**Table 4:** **Definition of genotype-specific metabolic models derived from bna572+**.

Genotype specific biomass component assembly (3231, 3170)	Biomass synthesis reaction (Biomasssynth_u) combining above components and free metabolites was modified according to Table 1. Triacylglycerol assembly reaction (TAGsynth_c) and Triacylglycerol degradation reaction (Lipase_c) according to fatty acid composition (Table 1)
General biomass component assembly reactions	Protein assembly reaction (Protsynth_c), DNA assembly (DNAsynth_u), RNA assembly (RNAsynth_u), Starch synthesis (starchsynth_p), Cell wall synthesis (CWsynth_a)^a^
Growth rate	Ex_biomass_u = 0.0725 mg/h^a^
Maintenance respiration	ATPdrain_c = 2.79 μmol/h^a^
Primary objective function	Minimize the sum of nutrient and light influxes. Minimize − 1^*^ (Ex_ph_t + Ex_glu_a + Ex_ala_a + Ex_asn_a + Ex_gln_a + Ex_fru_a + Ex_glc_a + Ex_sucr_a + Ex_no3_a + Ex_nh4_a + Ex_so4_a + Ex_pi_a)^a^
^13^C-derived flux constraints	See Table 2
Light flux	With addition of ^13^C-constraints (Table 2) light influx (Ex_ph_t) is predicted by optimization. The values of −4.541 and −5.079 μmol/h for 3231 and 3170, respectively, are similar to values reported by Hay and Schwender (2011a). For optimizations where ^13^C-constraints are omitted, Ex_ph_t is constrained to these values, respectively
Reactions knocked-out	Reactions of cytosolic OPPP not present^b^: TKT1_c, TKT2_c, TALDO_c
	Nutrients not available in growth medium are inactivated: Ex_asn_a, Ex_glu_a, Ex_fru_a, Ex_no3_a, Ex_nh4_a
	Artificial exchange reaction used in model reconstruction: Ex_atoco_p
	3′5′- hydroxylated flavonols^c^: myricetinsynth_c, phfsynth_c, dhmsynth_c, myricetinsynth2_c, dhmsynth2_c
Added Reactions^d^	Plastidic NAD-malate dehydrogenase (MDHnadh_p), Cytosolic malic enzyme, (ME_c)
Metabolites not forced to balance	h[c], h[m], h[p], h[x], h2o[c], h2o[l], h2o[m], h2o[p], h2o[x], sucr[e], glc[e], fru[e], gln[e], glu[e], ala[e], asn[e], no3[e], nh4[e], pi[e], so4[e], biomass[e], ph[e], o2[e], co2[e]
Limits on flux capacity	+/− 1000 μmol/h

*Detailed data see Table S1Q*.

a *Based on Hay and Schwender (2011a)*.

b *The GPR associations have been revised to remove cytosolic OPPP, but the reactions still exist in the SBML*.

c *3′5′- hydroxylated flavonols not found in B. napus*.

d *Reactions not yet formally added to the reconstruction*.

#### Confidence scores

Confidence scores were derived to quantify the level of biological evidence for reactions in the network (Tables S1B,E). The scores include information e.g., on evidence by biochemical characterization of the enzyme complement in isolated plastids of developing rapeseed embryos as well as evaluate reactions and GPR associations against multi-omics datasets, databases, and other organism-specific literature (see Materials and Methods). The scores given ranged between zero and four. Eighty percentage of all reactions had a score of at least 3.

#### Availability of Bna572+ reconstruction and models

Bna572+ is under continuous development and the version presented here and being used in simulations is dated February 18, 2014 (see model ID in File S1). Bna572+ is available in COBRA-compatible SBML (Hucka et al., 2003). MIRIAM Uniform Resource Identifiers (identifiers.org) (Juty et al., 2012) were used to define annotations. For practical usability, we include a test script (File S3) for the COBRA toolbox (Schellenberger et al., 2011b). Prior to use, the user needs only to install MATLAB and LibSBML (Bornstein et al., 2008). We also provide a Python script which can be used to translate the database's Excel worksheet into SMBL (File S2) for use with alternative programs such as COBRApy (Ebrahim et al., 2013).

### Analysis of metabolic network structure

**Figure 2:**
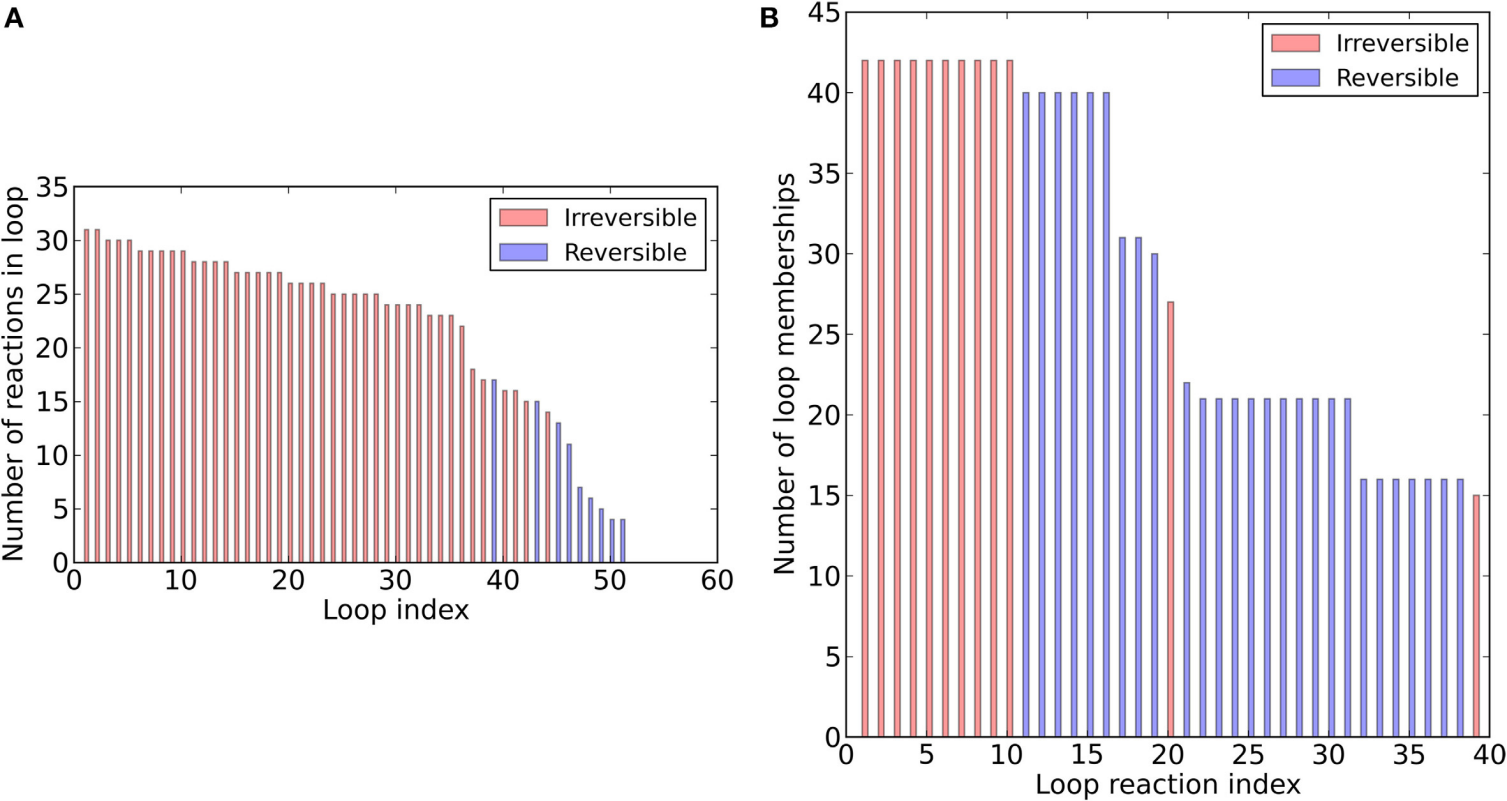
**Loop topology of bna572+**. Distribution of loop size **(A)** and loop membership **(B)**.

To assess the structure of the revised metabolic network reconstruction, we quantitatively analyzed some topological features. First, we assessed the degree of continuity of internal reactions with respect to inputs and outputs. In the unconstrained metabolic network, every reaction can carry flux at steady-state, i.e., there are not any blocked reactions (Table 3). This result reflects the fact that our pathway-by-pathway approach to reconstruction intentionally prevents dead ends in the metabolic network. Next, to analyze enzyme subsets, we randomly sampled the unconstrained network and identified correlated reactions sets, i.e., groups of reactions that always operate in fixed flux proportions. Using this approach, we identified 73 reaction subsets (Table S1B) ranging between 2 and 183 reactions large. Subset 1 is the largest and contains all the reactions going to biomass synthesis. Finally, we determined the loop topology of bna572+ (Figure 2, Table S1I). For these computations, instead of the unconstrained network, we used the photoheterotrophic-specific constraints with fixed light exchange flux (Table 4). Thirty nine reactions participate in loops (Figure 2B), i.e., thermodynamically infeasible internal cycles that result in unrealistic flux predictions. Extreme pathway analysis with Expa (Bell and Palsson, 2005) enumerated 51 loops (type III pathways) between 4 and 31 loop reactions in length (Figure 2A). Nine of these loops are reversible, i.e., they can carry flux in both directions. Loop reactions have membership with 15–43 loops. Due to these cycles the solution space was in part unbounded in FVA simulations (Table S1J). The loop reactions mostly belong to highly compartmentalized central intermediary metabolism. Mainly reactions of the subsystem categories of glycolysis, of citric acid cycle and carboxylic acid metabolism and intracellular transport reactions connecting the compartments cytosol, plastid, and mitochondrium are present. Hence, loops are a major problem predicting the feasible flux space in central carbon metabolism, motivating the use of the loopless algorithm.

The loopless algorithm involves mixed integer linear programming which can be computationally demanding. Therefore, we evaluated the computational performance of the loopless algorithm (Table S1M). The time to complete loopless FVA across all network reactions was 19–27 min. In contrast, FVA without the loopless algorithm took less than 1 min. Hence, loopless FVA takes significantly longer to compute. This is especially true for reactions with loop bounds, which took 6–10 times longer to calculate (Table S1M). Importantly, since loopless FVA offers no advantage for non-loop reactions, it makes sense in terms of computation time, to limit the algorithm to loop reactions. This technique is particularly useful given that the solver can get stuck, even on non-loop reactions (Table S1N, genotype 3170 no ratios). Bypassing non-loop reactions, we could bring the computation time for loopless FVA down to 7–9 min.

### Analysis of new subsystems (reaction 572+)

To characterize the predicted metabolic flux for the new subsystems of bna572+, we compared the newly reconstructed pathways and mapped flux variability analysis (File S5). Analysis of alternative biosynthetic pathways indicated which routes are predicted to carry flux with respect to the optimal flux space. For the vitamin E subsystem, tyrosine or phenylalanine contribute to the formation of the polar chromanol ring of alpha-tocopherol. For the isoprenoid pathways, the 5-C isoprenoid precursor isopentenyl diphosphate is produced by the non-mevalonate pathway, while the mevalonate pathway is predicted to not be used. Lastly, sinapate is predicted to be synthesized via conventional free phenylpropanoid acid biosynthesis, not the alternative route via shikimate O-hydroxycinnamoyltransferase (HCT) (#661). Since we showed that none of these reactions are blocked (Table 3), our analysis suggests that the unused pathways do not carry flux because they are suboptimal relative to alternative routes.

### Verification of gene expression

To verify the expression of genes in bna572+, we integrated developing embryo-specific gene expression data. Of particular value are 11464 *Arabidopsis* gene orthologs reported to be expressed in developing *B. napus* embryos (Troncoso-Ponce et al., 2011). Using this data set, we can validate the expression of 744 of the 966 genes in bna572+ (77%). By using proteome data of embryo plastids (Demartini et al., 2011), 10 more genes in bna572+ are validated. Leveraging the Boolean gene rule functionality of in total 545 GPR's (GPR, Table S1B) with the COBRA toolbox, we evaluated the reported presence/absence of transcripts and proteins (Table S1B). Based on transcriptome data, 523 (96%) of gene-associated reactions are present. If we also include the proteome data, this number increases to 526 (97%), i.e., 19 reactions with GPR lack support by gene expression. While the lack of expression diminishes to the overall confidence score, the 19 reactions for which gene expression was not found remained active in all model simulations reported—anticipating future improvements of data quality and methods. Closer inspection revealed that eight active reactions that were judged “non-present” were protein complexes consisting of seven or more different subunits (Sdh_m, FQR_p, NADH_m, ATP_p, PSI_p, PSII_p, PET_p). By Boolean logic defined in the GPR associations (AND), the lack of expression of only one subunit will cause an entire reaction to be called non-present. Also, expression of asparaginase (Asnase_c) and of phosphoglycolate phosphatase (PGLP_p), a step in photorespiration, was lacking. Missing expression of these reactions does not limit the confidence in the flux results since neither reaction is used in any of our simulations (Table S1J). For 8 additional reactions among Trp, Cys, purine, and phenylpropanoid synthesis (FOL_p, hfasynth_c, kdgsynth_c, narsynth_c, PAI_p, PUR_p, SAT_p, sinaldsynth_c) as well as two steps of the methylerythritol phosphate pathway (MEPCT_p, CMPkin_p) lack of expression might be more crucial.

### ^13^C-metabolic flux analysis and mapping Bna572+ to a ^13^C-model

**Figure 3:**
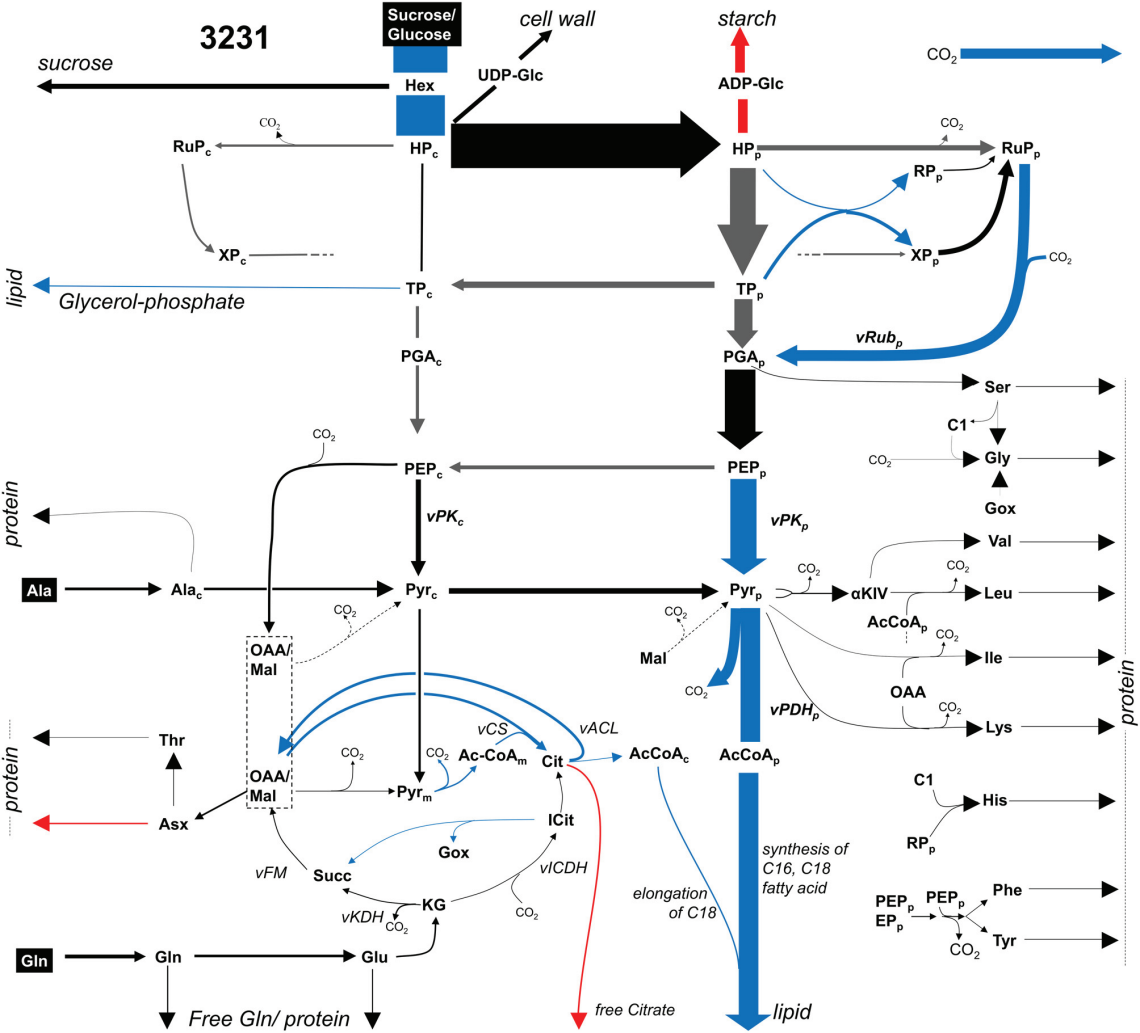
**Flux map for accession 3231 (^13^C-MFA)**. Flux distribution map showing glycolysis, pentosephosphate pathway, the TCA cycle and biosynthetic effluxes into protein, lipid and free soluble metabolites for accession 3231. Arrow thickness indicates carbon flux. Significant higher (lower) values in accession 3170 are marked in blue (red). Gray arrows: high statistical uncertainty (*SD* > 50% of flux value). Abbreviations: Metabolites (subscripts “p,” chloroplast; “c,” cytosol): AcCoA, acetyl-Coenzyme A; aKIV, 2-keto-isovalerate; C1, 5,10-methylene- or 5-formyl -tetrahydrofolate; Cit, citrate; GOX, glyoxylate; Hex, hexose, representing free hexoses and sucrose; HP, hexose phosphate; Icit, isocitrate; KG, ketoglutarate; OAA, lumped pool of subcellular pools of oxaloacetate and malate; PEP, phosphoenol pyruvate; PGA, 3-phospho glycerate; Pyr, pyruvate; RP, ribose 5-phosphate; RuP, Ribulose 5-phosphate; Succ, succinate; TP, triose phosphate (dihydroxyacetone phosphate, glyceraldehyde 3-phosphate); XP, xylulose 5-phosphate. Reactions: vACL, ATP:citrate lyase; vCS, citrate synthase; vFM, fumarase, malate dehydrogenase; vICDH, isocitrate dehydrogenase; vPDH_p, plastidic pyruvate dehydrogenase; vPK_p, plastidic pyruvate kinase; vRub_p, ribulose bisphosphate carboxylase.

Embryos of two *B. napus* accessions 3231 and 3170 were grown photoheterotrophically in culture under identical conditions with ^13^C-labeled glucose and sucrose as tracers for ^13^C-MFA (see Materials and Methods). Table S2B summarizes the network definition in relation to bna572+. In the ^13^C-MFA network, reactions are derived mainly by lumping successive or parallel reactions that can be represented by a common transition of carbon atoms and by lumping of metabolite pools (Schwender, 2011). The mapping relations are formalized in Table S2C. A ^13^C-MFA model with 128 reactions, comprising 647 carbon transitions in central metabolism was modeled (Materials and Methods). From the results of flux parameter fitting for the two genotypes, flux differed significantly between the genotypes for 13 reactions (Table S2G). Figure 3 gives an overview. As to be expected for the genotype higher in oil (3170), flux into lipid synthesis is increased. The increased demand in biosynthetic precursors is met by increased flux of plastidic pyruvate kinase (vKPp), pyruvate dehydrogenase (vPDHp), and efflux of triose-phosphate (synthesis of glycerol). The uptake of sugar substrate is increased as well, indicating an increased sugar to lipid conversion. An increase in citrate synthase (vCS) and ATP:citrate lyase (vACL) flux in accession 3170 can be explained by higher demands for cytosolic fatty acid elongation.

Next we determined flux ratio- and flux constraints to be applied to the large scale model. With the initial model configuration the flux state of free fluxes is defined by three free uptake fluxes and ten inner free fluxes. This configuration was replaced by one with seven flux ratios and five free net fluxes (vME_c, vME_p, vPPT, vG6PDH_p, vAla_bm) (Table S2H). Two free fluxes (vME_c, vME_p) were close to zero in both genotypes and thus the equivalent reactions in bna572+ (ME_c, ME_p) were constrained to zero. All resulting constraints are listed in Table 2.

### Genotype-specific simulations of Bna572+

**Figure 4:**
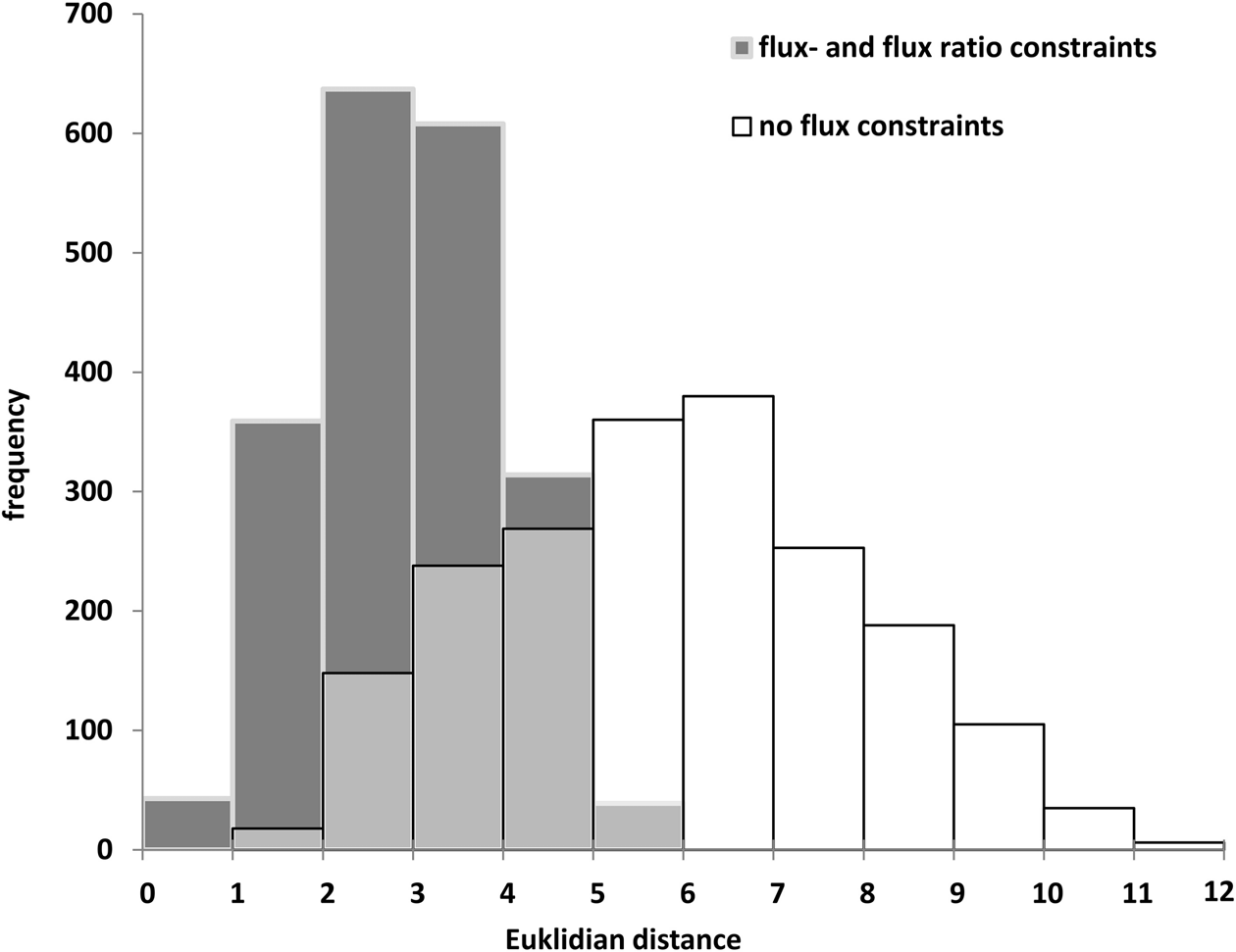
**Random sampling of loopless solutions showing the effect of flux- and flux ratio constraints**. Genotype 3170. Euclidean distance between random sampled flux vectors, projected onto the ^13^C-MFA model, and ^13^C-MFA flux estimates.

The biomass composition of the two *B. napus* accessions 3231 and 3170 differed principally in two components. Accession 3170 is 10% (w/dw) higher in lipid content and 3231 is by 6% higher in starch content (Table 1). These differences in biomass composition were used to derive genotype specific models from bna572+. Transformation of the bna572+ reconstruction into a metabolic model requires addition of physiological constraints, which include biomass composition-derived fluxes, biomass accumulation rate, fixed maintenance respiration rate, and inactivation of exchanges for external metabolites not available in the medium. Details on constraints imposed on bna572+ when simulated under COBRA can be found in Table 4. As before, flux balance analysis methods were carried out with photoheterotrophic constraints under the primary objective of minimization of total molar substrate uptakes (Table 4, Hay and Schwender, 2011b). Essentially, the primary optimization adjusts the model to a carbon balance that is derived from the ^13^C-tracer experiments performed under photoheterotrophic conditions (Table 2, r7). The light flux affects the total carbon balance since photosynthetic electron transport is a source for carbon reduction. The primary optimization serves to adjust the light flux accordingly. The light fluxes hereby obtained for both accessions (Table 4) are similar to the one formerly obtained based on constraining the model with independently measured carbon balances (Hay and Schwender, 2011a). In addition to the carbon balance constraint, we applied six genotype specific flux ratio- and two reaction knock-out constraints (Table 2), based on the flux distribution in central carbon metabolism as inferred by ^13^C-MFA (Table S2G). Flux states resulting from these model configurations were then assessed to characterize the effect of ^13^C-flux and flux ratio constraints on the size of the optimal flux space. First we assessed the variability type distribution. FVA produces bounds for all reactions which can be categorized according to being “variable” (difference between lower and upper bound), “non-variable” (both bounds are equal and non-zero) and “never used” (both bounds are zero) (Hay and Schwender, 2011b). One might expect that addition of ^13^C-constraints reduces the flux space by reducing the number of variable reactions. Remarkably, for both accessions the total number of 89 variable reactions is unchanging, with or without ^13^C-flux ratio constraints (Table S1K). Instead, reduction of flux space can be seen when assessing the total range of flux variability (sum of size of all flux intervals across bna572+). Accordingly, presence of ^13^C-derived flux constraints reduce the loopless solution space by 53 and 55% for accessions 3231 and 3170, respectively (Table S1M). We also applied random sampling of loopless flux states within the boundaries given by the flux variability intervals (Table S1O). In particular, the random sampling was done in projection of bna572+ onto the ^13^C-MFA network. For each of the sampled states, the flux ratios originally derived from 13C-MFA were reproduced (Table S1O). This verifies the computational process in-between the ^13^C-MFA and bna572+. In addition, the Euclidian distance between the random sampled flux states and the original ^13^C-MFA derived flux states reduces substantially by imposing the ^13^C-derived constraints (Figure 4). This again demonstrates the reduction in flux space. Closer inspection of FVA projections onto the ^13^C-MFA network shows that with ^13^C-MFA constraints 15 reactions have flux variability (Table S1L). The 15 reactions are part of the glycolysis and PPP reactions present in parallel in the cytosol and in the plastid compartment. These represent free fluxes in the ^13^C-MFA model that had relatively high statistical uncertainty and therefore were not transferred (see Materials and Methods).

### Differential flux variability and reaction responsiveness in *Brassica napus* genotypes

To better understand the genotype-specific differences in carbon allocation to embryo biomass during development, we compared the metabolic phenotypes of *B. napus* accessions 3231 and 3170 predicted with FVA. The change in predicted flux between the high oil genotype 3170 and 3231 was qualitatively characterized by assigning each reaction a responsiveness type (Table S1J), the frequencies of which are tabulated in Table S1K and summarized in Table 5. Applying the loopless constraints, there are 27 more responsiveness predictions than without. This is because multiple reactions participate in loops in both genotype models and therefore have undefined (infinite) flux bounds, which become identifiable with added loopless constraints. The reactions that become identifiable are located mainly in central metabolism. Overall, with the loopless algorithm and ^13^C-MFA derived constraints, 351 oil- and 41 starch-responsive reactions were identified (Table 5). Via GPR associations, this information can be integrated with gene expression data, as explored in our companion paper (Schwender et al., 2014).

**Table 5:** **Distribution of predicted reaction responses to explain the difference between high oil/low starch genotype 3170 and low oil/high starch genotype 3231**.

**Responsiveness^a^**	**L^+^ R^−^**	**L^+^ R^+^**	**L^−^ R^−^**	**L^−^ R^+^**
T	335	351	306	333
S	15	41	15	43
T/S^b^	2	17	2	4
N	319	262	321	264
Undefined^c^	0	0	27	27

*Flux variability determined with or without loopless algorithm (L^+^/L^−^) and with or without flux constraints from ^13^C-MFA (R^+^/ R^−^). All results see Table S1J*.

a *“T,” flux bound magnitude higher in high lipid (Triacylglycerol) genotype 3170; “S,” flux bound magnitude higher in high starch genotype 3231; “N,” no change*.

b *Two flux bounds of opposite responsiveness*.

c *Change in flux bound magnitude undefined for unbounded flux solutions*.

We further characterized the metabolic phenotypes by subsystems (Table S1P). Lipid biosynthetic pathways increase in flux in the high oil genotype, as indicated by their high enrichment for oil-responsiveness. The same was true for the light reactions of photosynthesis, Calvin cycle, Rubisco shunt, oxidative pentose phosphate pathway (OPPP), and various amino acid biosyntheses (the high-oil phenotype is also slightly higher in protein; Table 1). Unexpectedly, in a portion of the beta-oxidation subsystem flux was up upregulated in the high oil genotype (#rxns 413–437, 466–468, 519–20). This observed pattern did not involve lipase (#133), but constitutes a futile cycle of fatty acid synthesis and degradation of relatively small magnitude, which must be suboptimal. A different set of subsystems was highly enriched for starch-responsiveness. Up-regulated reactions predicted in the high-starch genotype include starch biosynthesis, pyrophosphate metabolism, and oxidative phosphorylation. Glycolysis and the citric acid cycle were divided between starch and oil responsiveness, with mitochondrial citrate synthase (#37) and pyruvate dehydrogenase (#541–543) correlated with starch, and the plastidic isoform of pyruvate dehydrogenase (#480–482) being oil-responsive. These relationships are further explored in our companion paper (Schwender et al., 2014).

### Limitations

We have demonstrated here how the solution space in the large scale model bna572+ can be further constrained based on experimental data, namely ^13^C-MFA derived fluxes, together with elimination of unboundedness based on the loopless-COBRA algorithm. However, interpretation in particular of qualitative flux variability types and of flux responsiveness has to be made carefully, bearing in mind that small differences might not be statistically significant. While the ^13^C-MFA model results in flux estimates with statistical uncertainty measures, the constraint-based approach produces flux estimates without statistics. We address this problem in part by not including in the CB model any flux estimates from MFA that are statistically poorly defined. Yet, the biomass composition data for the two compared *B. napus* accessions were defined in the CB-model using the commonly used biomass equation - without statistical measures. Between the two accessions 3170 and 3231 there is a very small difference in protein content (Table 1). On the level of ^13^C-Flux analysis, this difference does translate into statistically insignificant differences in amino acid and protein biosynthesis fluxes, while in the CB-model, the same small differences are judged “TAG responsive” by the criteria used to determine quantitative responsiveness. Furthermore, some pattern of pathway usage being observed in response to imposition of ^13^C-constraints onto genotype specific bna572+ cannot easily be explained. Imposing genotype 3170 derived ^13^C-constraints resulted in a small scale energetically wasteful futile cycle of fatty acid synthesis and degradation. This is unexpected since the ^13^C-MFA model does not contain beta-oxidation. Altogether this means that interpretation of the small flux responses should be done with caution and a comprehensive statistical treatment of CB-predictions is warranted.

## Discussion

### Relevance of Bna572+

Bna572+ extends the previous predictive model of *B. napus* seed development, bna572 (Hay and Schwender, 2011a,b; Schwender and Hay, 2012; Borisjuk et al., 2013). Lacking a complete and fully annotated *B. napus* genome, our model is referenced to the *Arabidopsis* genome. Such hybrid models that are indirectly derived by orthology to *Arabidopsis* are not uncommon, including models of C4 and CAM plants (De Oliveira Dal'molin et al., 2010b; Cheung et al., 2014). In our case, using the *Arabidopsis* genome as a proxy is highly justified given the close relationship between *B. napus* and *Arabidopsis* within the *Brassicaceae* (Al-Shehbaz et al., 2006). In an accompanying paper (Schwender et al., 2014) we explore how, using the *Arabidopsis* genome as a proxy, *B. napus* hiseq transcriptome sequencing data can be integrated with bna572+.

Bna572+ is relatively small compared to reconstructions of *Arabidopsis* which were derived in a top-down manner starting with a set of genome predicted metabolic genes (Table 6). Given the complex subcellular compartmentation of metabolism in plants, model reconstruction aimed at comprehensive representation of genome encoded reactome is not necessarily the only meaningful strategy for network reconstruction, in particular since even the *Arabidopsis* genome is largely underexplored in terms of transport proteins in primary and secondary metabolism. It appears that in genome-scale *Arabidopsis* reconstructions a high percentage of reactions is associated with the cytosol (Table 6). This indicates that compartmentation of pathways tends to be a major challenge in model reconstruction, since the cytosol is typically the default localization in first draft reconstructions derived from uncompartmentalized pathway databases. An exeption to this retention of cytosolic localization is seen in the Mintz-Oron model (Table 6). However, in this case, all intracellular transport reactions are entirely computationally inferred based on a principle similar to gap filling (Mintz-Oron et al., 2012), which might be unrealistic. Here we favor an expandable bottom-up reconstruction process that is guided by adding metabolic functions according to major biomass components relevant to the studied tissue and by emphasizing compartmentation over comprehensiveness. Compartmentation of specific pathways can often be defined from original expert domain literature, which often implies which metabolites might be transported across membranes without necessarily identifying specific transporter proteins. Altogether, bna572+ is a valuable resource for computational and experimental scientists in the *Brassicacea* community and foreseeable community-driven efforts for *Arabidopsis* consensus models.

**Table 6:** **Basic characteristics of *Arabidospis thaliana* pathway databases and metabolic reconstructions**.

**Model name/References**	**Primary sources for metabolic reconstruction**	**Genes**	**Unique reactions**	**Subcellular compartmentation**
***ARABIDOPSIS* PATHWAY DATABASES**				
AraCyc^a^	N/A	3338^b^	1966^c^	Very little information on compartmentation and intracellular transporter
KEGG	N/A	3349^d^	1715^e^	Very little information on compartmentation and intracellular transporter
**TOP-DOWN METABOLIC RECONSTRUCTIONS**				
Poolman et al., 2009	AraCyc (ver. 4.5)	N/A	1406	Not compartmentalized; No inter-organellar transport
Cheung et al., 2013	AraCyc (ver. 9.0)	N/A	2769	2036 (74%) in cytosol^f^, 417 (15%) in other compartments^f^; 192 (7%) inter-organellar transport;
AraGEM; De Oliveira Dal'molin et al., 2010a	AraCyc (ver. 5.0), KEGG	1419	1567	1265 (75%) in cytosol^f^, 317 (19%) in other compartments^f^; 83 (5%) inter-organellar transport
iRS1597, derived from AraGEM; Saha et al., 2011	AraGEM	1597	1798	1533 (85%) in cytosol^f^, 162 (9%) in other compartments^f^; 81 (4.5%) inter-organellar transport (no sym- or antiport);
Mintz-Oron et al., 2012	AraCyc, KEGG	1791^g^	2942	604 (20%) in cytosol^f^, 1584 (53%) in other compartments^f^; 654 (22%) inter-organellar transport: connections between cytosol and 6 other compartments inferred by algorithm (no sym- or antiport)
**BOTTOM-UP METABOLIC RECONSTRUCTIONS**				
Bna572; Hay and Schwender, 2011a	KEGG	558	572	206 (36%) in cytosol^f^, 336 (59%) in other compartments^f^; 103 (19%) inter-organellar transport; 28 sym- or antiport
Arnold and Nikoloski, 2014	Aracyc (ver. 11.5)	628	549	100 (18%) in cytosol^f^, 217 (40%) in other compartments^f^; 264 (45%) inter-organellar transport, none with GPR, 28 sym- or antiport
Bna572+ (this publication)	KEGG and Aracyc	958	669	162 (24%) in cytosol^f^, 359 (54%) in other compartments^f^; 111 (17%) inter-organellar transport; 40 of them with GPR, 28 sym- or antiport

a *Data from: ftp://ftp.plantcyc.org/Pathways/Data_dumps/PMN8_July2013/aracyc_pathways.20130709*.

b *Unique TAIR locus ID's*.

c *Unique reaction ID's*.

d *Genes associated with an EC number (http://rest.kegg.jp//link/ec/ath) or reaction id (http://rest.kegg.jp//link/rn/ath) (Accessed August 6, 2014)*.

e *Unique reactions associated with genes http://rest.kegg.jp//link/rn/ath (Accessed August 6, 2013)*.

f *Reactions that take place exclusively in one compartment*.

g *Unique TAIR IDs in supplement sd02 (Mintz-Oron et al., 2012)*.

### Importance of standardization

According to published guidelines (Thiele and Palsson, 2010), bottom-up genome-scale reconstruction is a laborious task that involves labor and time intensive manual curation of multi-omics data used in systems biology. Various software platforms exist to automate initial steps in the reconstruction process (Hamilton and Reed, 2014). For example, the SuBliMinaL (Swainston et al., 2011) toolbox of path2models (Buchel et al., 2013) in the BioModels database outputs a draft reconstruction of 1672 reactions and 2032 genes for *Arabidopsis*, leaving the arduous task of model refinement to the reconstructionist. Other platforms have been designed for model building and curation. For example, the rBioNet extension for COBRA offers a convenient graphical user interface for quality control and assurance (Thorleifsson and Thiele, 2011). While clearly there are many options for the reconstructionist, the workflow we report here offers the familiarity of Excel spreadsheets and hence knowledge database flexibility. It is along the lines of the xls2model function that was developed for COBRA (Schellenberger et al., 2011b). The difference is that our platform requires the user to write SBML as a model intermediate in a Python scripting environment. Importantly, COBRA-compliant SBML, of the kind we provide here (File S1), is the mainstay of metabolic systems biology by making predictive metabolic models publishable and distributable for actual use.

With the COBRA standard, a wealth of methods is now applicable to bna572+ for systems-level analysis and metabolic engineering. For example, gene deletions in the reaction network could be characterized as is commonly done with microbes (Monk and Palsson, 2014), or OptKnock (Burgard et al., 2003) could be used to predict non-intuitive knockout strategies for the redesign of biomass composition.

### Tissue specific vs. genome scale model

Genome-scale models of higher organisms typically represent global metabolic reconstructions. To simulate cell-type or tissue specific metabolism, models are being integrated with information on tissue specific gene expression (Machado and Herrgard, 2014). For example, proteome data have been used to generate tissue specific models from an *Arabidopsis* genome scale model (Mintz-Oron et al., 2012). Bna572+ was derived as a bottom-up reconstruction already with a developing seed in mind, but now also allows integration of transcriptomic data. This means transcriptomic data can be used to validate the model or to further constrain the flux space. A next step would be to identify which of the several mathematical methods being available (Machado and Herrgard, 2014) might be best suited for our specific system.

### Shrinking the solution space using ^13^C-constraints and loopless algorithm

Here we integrated ^13^C-MFA and CB-modeling. ^13^C-MFA results in empirical flux data based on the use of ^13^C-tracers, while CB-modeling is a rather predictive modeling approach. A principal problem commonly encountered for CB-modeling is that of the missing of constraints (Reed, 2012) and the ambiguity caused by prediction of multiple alternative flux distributions. This applies in particular to highly compartmentalized plant cells. The boundaries of this solution space can be explored by FVA (Mahadevan and Schilling, 2003) or by random sampling (Wiback et al., 2004). If objective functions are used that give unique solutions, internal predicted flux states can differ substantially from ^13^C-MFA-derived empirical flux data. In order to improve the predictive power of CB-modeling a variety of approaches have been developed in trying to further narrow down the most likely biologically relevant flux solutions (Park et al., 2010; Lerman et al., 2012; Reed, 2012). In plants, alternative solutions and ambiguity have been addressed for example by the choice of objective function(s) (Cheung et al., 2013), integration of gene expression data (Topfer et al., 2013), FVA (Hay and Schwender, 2011b) and simplification by network projection (Hay and Schwender, 2011a). Another resource for refining flux predictions based on experimental data is the integration of ^13^C-MFA data. Flux ratio constraints have been used to better predict experimentally determined fluxes (Hay and Schwender, 2011b; Cheung et al., 2013). However, only a few ratios were used in these studies. In this paper, most of the information about the flux determination in ^13^C-MFA has been transferred to the large scale model via seven flux ratios and two flux constraints (Table 2). As a result, the predictive potential of our model increased substantially (Figure 4).

Another major problem with FVA is the prediction of flux states involving net flux around a closed cycle of internal reactions. In this study, the predictive power of bna572+ was severely challenged by such thermodynamically infeasible loop flux (Figure 2, Table 5). The loopless COBRA algorithm (Schellenberger et al., 2011a) proved to be a good solution to eliminate these problematic cycles because, in contrast to other methods (Muller and Bockmayr, 2013), it did not require any information about metabolite concentrations or thermodynamic parameters. Surprisingly, so far there has been limited use of loopless COBRA in plant systems biology (Mintz-Oron et al., 2012), even though it is implemented in its namesake toolbox. As shown by the genotype response of FVA reported here, we expect the loopless constraints to improve the predictive power of *in silico* biomass component tradeoffs (Schwender and Hay, 2012).

### Comparison of two genotypes

In this study we compared two *B. napus* accessions which differed mainly in lipid and starch content of *in-vitro* cultivated embryos. By using constraints derived from the flux estimation by ^13^C-MFA for two genotype specific FVA models, we expanded on the concept of *in silico* biomass component tradeoffs (Schwender and Hay, 2012) and could show that the flux solution space is being reduced. Starch biosynthesis responsiveness was predicted for the low-oil genotype as expected given the biomass composition. Likewise, for the high-oil genotype 3170, increase in flux through fatty acid synthesis, elongation, desaturation, triacylglyerol synthesis, and amino acid/protein biosynthesis were predicted. A few of the reactions of these subsystems can be validated with correlations of gene expression (Table 4 in Schwender et al., 2014).

### Conflict of interest statement

The authors declare that the research was conducted in the absence of any commercial or financial relationships that could be construed as a potential conflict of interest.
